# Human Trichinosis after Consumption of Soft-Shelled Turtles, Taiwan

**DOI:** 10.3201/eid1512.090619

**Published:** 2009-12

**Authors:** Yi-Chun Lo, Chien-Ching Hung, Ching-Shih Lai, Zhiliang Wu, Isao Nagano, Takuya Maeda, Yuzo Takahashi, Chan-Hsien Chiu, Donald Dah-Shyong Jiang

**Affiliations:** Centers for Disease Control, Taipei, Taiwan (Y.-C. Lo, C.-H. Chiu, D.D.-S. Jiang); National Taiwan University College of Medicine, Taipei (C.-C. Hung); Department of Health, Taipei (C.-S. Lai); Gifu University Graduate School of Medicine, Gifu, Japan (Z. Wu, I. Nagano, Y. Takahasi); University of Tokyo, Tokyo, Japan (T. Maeda)

**Keywords:** Trichinella, trichinosis, zoonoses, parasites, soft-shelled turtles, Taiwan, dispatch

## Abstract

In 2008, an outbreak of human trichinosis associated with ingestion of raw soft-shelled turtles was identified and investigated in Taiwan. The data suggested that patients were likely infected with *Trichinella papuae.*

Trichinosis is a zoonotic disease caused by species of the nematode *Trichinella*. In eastern Asia, human trichinosis has been reported in the People’s Republic of China, Japan, and Korea (*1*–*4*). Trichinosis among humans and animals has not been reported in Taiwan (*1*,*5*,*6*). Although the major source of human infection is the meat of mammals, reptiles recently have been found to serve as hosts for certain *Trichinella* species. *T. zimbabwensis*, detected in the muscles of Nile crocodiles (*Crocodylus niloticus*) in Zimbabwe in 1995, is the first species of *Trichinella* found in a reptile host naturally infected with *Trichinella* (*7*). No human infection has been documented. Another species, *T. papuae*, was detected in a farmed saltwater crocodile in Papua New Guinea in 2004 (*8*). A trichinosis outbreak in humans caused by *T. papuae*, associated with eating wild boar meat, occurred in Thailand (*9*). Trichinosis in humans related to consumption of reptile meat was first described in Thailand; the source was turtle and brown lizard meat (*10*). We report an outbreak of human trichinosis in Taiwan in which eating raw soft-shelled turtles (*Pelodiscus sinensis*) was the suspected mode of infection.

## The Study

In July 2008, four teaching hospitals in northern Taiwan consecutively reported to the Department of Health of Taipei City Government (DHTCG) and the Centers for Disease Control (CDC) 8 patients in 2 groups in whom fever, myalgias, and eosinophilia of unknown cause developed after they shared a common food source in May 2008. Group A, comprising 20 Taiwanese, participated in a festive meal in Taipei City at a Japanese food restaurant, and were served raw meat, blood, liver, and eggs of 3 of the 5 soft-shelled turtles provided by the host, a supplier of soft-shelled turtles. The other 2 soft-shelled turtles were refrigerated at 4°C and served at the same restaurant to a group of 3 Japanese customers (group B) 6 days later.

DHTCG and Taiwan CDC jointly investigated this outbreak. The restaurant had never previously served raw or undercooked soft-shelled turtles. Restaurant patrons other than those in groups A and B did not eat raw or undercooked soft-shelled turtles. Five of the 20 Taiwanese in group A and the 3 Japanese in group B exhibited signs and symptoms 1–3 weeks after eating at the restaurant and were defined as case-patients (Table 1). The 15 asymptomatic persons were defined as controls.

**Table 1:** Clinical characteristics and results of serologic assays of 8 case-patients with *Trichinella* infection, Taiwan, 2008

Patient no./group	Age, y/ sex	Incubation period, d	Symptoms	Eosinophils, cells/μL	Acute-phase titer†			Convalescent-phase titer‡	
					*T. spiralis*	*T. pseudospiralis*		*T. spiralis*	*T. pseudospiralis*
1/A	60/M	7	Fever, myalgia, malaise	6,825	25,600	25,600		51,200	51,200
2/A	52/M	6	Fever, myalgia, malaise, periorbital swelling, leg swelling	3,815	800	400		51,200	51,200
3/A	57/M	14	Myalgia, malaise, trismus, tremor	2,713	NA*	NA		12,800	12,800
4/A	57/F	8	Fever, chills, dyspnea, myalgia, malaise, trismus, tremor, periorbital swelling	5,055	NA	NA		51,200	51,200
5/A	62/M	15	Fever, myalgia, malaise	1,421	3,200	1,600		51,200	51,200
6/B	52/M	8	Fever	4,461	12,800	6,400		51,200	25,600
7/B	57/M	7	Fever, myalgia, leg swelling, periorbital swelling, skin rash	8,505	12,800	12,800		25,600	12,800
8/B	47/M	8	Fever, myalgia	NA	NA	NA		25,600	25,600

*NA, not applicable. †Weeks 3–5 postexposure. ‡Weeks 7–9 postexposure.

The most common symptoms were myalgia (88%), fever (88%), malaise (63%), and periorbital swelling (38%). Seven case-patients whose blood was analyzed all had eosinophilia and increased serum creatine phosphokinase and alanine aminotransferase levels. Five case-patients were hospitalized. Two patients underwent extensive serologic testing for helminths by ELISA, including tests for *Dirofilaria immitis, Toxocara canis, Ascaris lumbricoides, Anisakis* spp*., Gnathostoma spinigerum, Strongyloides stercoralis, Paragonimus westermanii, Paragonimus miyazakii, Fasciola hepatica, Clonorchis sinensis, Spirometra erinacei, Taenia solium, and Trichinella* spp. Both patients had weakly positive results for *A. lumbricoides, G. spinigerum,* and *S. stercoralis* and strongly reactive results for *Trichinella* spp.

Serum samples from 5 patients during the acute phase (3–5 weeks postexposure) and from all 8 patients during the convalescent phase (7–9 weeks postexposure) were sent to the Department of Parasitology, Gifu University, Gifu, Japan, for *Trichinella* serologic diagnosis, with ELISA and immunohistochemical staining. Of the 15 controls, none consented to give a blood sample. Briefly, the ELISA microtiter plates were sensitized with excretory-secretory (ES) antigen from *T. spiralis* or *T. pseudospiralis*, probed with a diluted human serum sample (1:200–1:6,400), and incubated with 100 μL of 1:10,000-diluted goat antihuman immunoglobulin G (Fab specific) peroxidase-conjugate (Sigma Chemical Co., St. Louis, MO, USA). Absorbance at 414 nm was monitored with a plate reader. All samples were analyzed in duplicate. The cutoff point was 3× the mean values of the A_414_ for the negative controls. Immunohistochemical staining was performed by incubating skeletal muscle tissues from *T. spiralis–*infected mice with the serum specimens (1:200 dilution) for 1 h at 37°C and processing the sections with the HistoStain SP kit (Zymed Laboratories Inc., San Francisco, CA, USA).

In both the acute and convalescent phases, all serum samples reacted to *T. spiralis* and *T. pseudospiralis* ES antigen and were positive in immunohistochemical staining (Table 1). The diagnosis of trichinosis was confirmed. Mebendazole or albendazole was prescribed for all patients, and their symptoms gradually resolved.

We conducted semistructured interviews with the 8 case-patients and 15 controls in both groups to learn which food items they had eaten at the restaurant. None had eaten raw or undercooked soft-shelled turtles before this outbreak (Table 2). In univariate analysis, consumption of raw soft-shelled turtle meat was strongly associated with infection (p = 0.003). Trichinosis developed in 8 (62%) of the 13 persons who ate raw soft-shelled turtle meat.

**Table 2:** Results of univariate analyses of selected food items in an outbreak of trichinosis, Taiwan, 2008*

Ingested food items	Case (n = 8)			Control (n = 15)		OR (95% CI)†	p value‡
	Ate	Did not eat		Ate	Did not eat		
Soft-shelled turtles							
Raw meat	8	0		5	10	–	0.003
Fried meat	6	2		14	1	0.21 (0.003–5.22)	0.269
Raw liver	7	1		8	7	6.13 (0.51–314.71)	0.176
Fresh blood	6	2		7	8	3.43 (0.40–43.28)	0.379
Raw eggs	7	1		10	5	3.50 (0.28–188.78)	0.369
Raw intestines	3	5		2	13	3.90 (0.32–56.52)	0.297
Cooked soup	7	1		13	2	1.08 (0.05–72.50)	1.000
Rice with cooked eel	7	1		15	0	–	0.348
Raw abalone	6	2		12	3	0.75 (0.07–11.43)	1.000

*OR, odds ratio; CI, confidence interval. †Significant at α = 0.05. ‡By Fisher exact test.

We performed an environmental study of the restaurant and the soft-shelled turtle farm. No leftover food was available from the restaurant for analysis. The soft-shelled turtles were bred artificially and hatched on a farm in Taiwan. They were fed only indigenous fish and shellfish. The farm used neither imported feed nor feed containing any mammals or reptiles. Microscopic inspection, with a meat-digesting method, of the soft-shelled turtles obtained from the farm 2 months after the outbreak did not show *Trichinella* spp. After the investigation, Taiwan CDC issued a press release to describe the outbreak and alert the public of the risk for trichinosis from eating raw or undercooked soft-shelled turtles.

## Conclusions

The incubation period, clinical features, and laboratory findings in this outbreak are similar to those of other reported trichinosis outbreaks associated with eating mammals (*11*,*12*). *T. papuae* and *T. zimbabwensis* are the most likely parasites causing this outbreak because of their ability to infect mammals and reptiles (*13*). The ELISA method has limited specificity because of cross-reactions with non-*Trichinella* helminths (*14*). Moreover, because of similar antigen patterns among all *Trichinella* spp., the antigens prepared with 1 species can be used to detect specific antibodies in patients infected with any species (*1*). Therefore, although we detected strongly reactive antibodies to *T. spiralis* and *T. pseudospiralis*, we could not determine the etiologic *Trichinella* sp. in this outbreak without parasitic diagnosis.

A recent study demonstrated that the 53-kDa recombinant proteins in larval ES products could provide species-specific antibody responses in *Trichinella*-infected mice (*15*). We assessed the absorbance at 414 nm with a 1:200-diluted serum sample in our patients by using the 53-kDa recombinant proteins expressed from 5 *Trichinella* species (*T. spiralis, T. britovi, T. nativa, T. pseudospiralis, and T. papuae*). Our preliminary results showed that convalescent-phase serum specimens from 6 of the 8 case-patients reacted most strongly to the 53-kDa recombinant protein of *T. papuae*. Although application of this method in species-specific human diagnosis requires further studies, the data suggest our patients were likely to be infected with *T. papuae.* Because we have not yet determined how soft-shelled turtles were infected by *T. papuae* in this outbreak, further investigation of the potential infectious source is warranted.

Persons in many parts of the world typically consume raw or uncooked reptile meat. Further investigations are urgently needed to assess the epidemiology of reptile trichinosis and the human risk for trichinosis from reptiles.
